# Decisional Conflict in Regarding Deinfibulation in Female Genital Mutilation/Cutting Patients: A Cross-Sectional Study

**DOI:** 10.1007/s10903-026-01916-w

**Published:** 2026-04-17

**Authors:** Shreya Chanda, Ryan L. Rahm-Knigge, Nicole Chaisson, Beatrice “Bean” E. Robinson, Anisa Hagi-Mohamed, Jennifer Jo Connor

**Affiliations:** 1 Medical School, University of Minnesota, Minneapolis, USA; 2 Eli Coleman Institute for Sexual and Gender Health, Department of Family Medicine and Community Health, University of Minnesota Medical School, University of Minnesota, Minneapolis, USA; 3 Department of Family Medicine and Community Health, University of Minnesota Medical School, University of Minnesota, Minneapolis, USA; 4 Maangaar Voices, Bloomington, USA

**Keywords:** Female genital mutilation/cutting, Deinfibulation, Decisional conflict, Somali healthcare, Sexual function, General gynecology

## Abstract

Many Somali female migrants experience Female Genital Mutilation/Female Genital Cutting (FGM/C) during childhood, a procedure that involves removal of the external female genitalia for non-medical reasons; most undergo infibulation, which involves narrowing of the vaginal introitus. FGM/C patients commonly undergo deinfibulation, a procedure that reopens the vaginal introitus. This study aimed to analyze factors that contribute to a participant’s decision to undergo deinfibulation using the Decisional Conflict Scale (DCS) and develop recommendations for healthcare providers to ease decisional conflict. This cross-sectional study recruited 300 Minnesotan Somali women with FGM/C. Participants were given a survey including a modified version of the DCS, which aimed to assess the amount and nature of decisional conflict faced by participants regarding deinfibulation. We found that 196 individuals were infibulated, of which 144 individuals (73.0%) were deinfibulated. The total Decisional Conflict Scale results indicated a moderate level of decisional conflict. The largest source of decisional conflict was regarding a lack of comprehensive understanding of the options surrounding deinfibulation. Timing of deinfibulation was found to be associated with DCS total score. The group that never underwent definfibulation had the most overall decisional conflict, while those that deinfibulated during pregnancy/childbirth had the least (*p* < .001). The results of this study indicate a knowledge gap and lack of health care provider (HCP) support for patients regarding deinfibulation, which contributes to decisional conflict in the population of Somali women considering deinfibulation. Further research on the efficacy of SDM principles on this topic is needed to guide clinical recommendations.

## Background

Shared decision making (SDM) is a collaborative decision-making approach by which patients and clinicians assess care options, evaluate benefits and harms of each, and select courses of action that integrate patients’ preferences [1]. The crux of SDM is about honoring patients’ personal preferences [2]. In this way, SDM is a method of patient care [3, 4] that can help reduce decisional conflict [5], especially in an obstetric and gynecological setting [6]. Decisional conflict is defined as the experience of uncertainty when facing a medical decision [7]. Contributing factors to decisional conflict include lack of information, shifting personal values, and feeling unsupported [8]. Interventions designed to lower decisional conflict can improve knowledge and healthcare provider (HCP)-patient communication [9]. However, evidence suggests that socially disadvantaged populations are at risk for exclusion from SDM. For example, SDM during childbirth for Black women in the United States (U.S.) is lower than White women [7]. Further, the use of interventions to reduce decisional conflict among socially disadvantaged populations has been understudied.

Refugees in the U.S. face additional barriers to SDM, including the impact of language and cultural differences on HCP-patient communication [10]. Additionally, practices not typically conducted in the U.S., such as female genital cutting (FGC), may lead to misunderstanding and othering of patients [11]. FGC, defined as modifications to female external genitals for non-medical purposes, impacts over 230,000 girls and women worldwide [12, 13]. It occurs predominantly during childhood in Africa and the Middle East. The World Health Organization defined four types of FGC; the most invasive type is infibulation (Type 3) which involves “narrowing of the vaginal orifice with creation of a covering by cutting and appositioning the labia minora and/or the labia majora, with or without excision of the clitoris” (p. 10) [14]. Infibulation is most commonly found in East Africa, particularly in Somalia, where the latest available estimates indicate over 90% have been cut and approximately two-thirds are infibulated [13]. Many Somali girls and women who have been infibulated have emigrated to neighboring countries (including refugee camps), European countries, and North American countries – with a particularly high population in Minnesota [15–17]. An important concern regarding SDM for women who have been infibulated is that HCPs may be unequipped to address health needs related to infibulation.

Meta-analyses have shown a wide range of experiences and morbidity levels with FGC, including several long-term complications such as increased risk of obstetric complications, dyspareunia, sexual dysfunction, urinary concerns, and infertility [18–21]. Patients who have been infibulated may undergo deinfibulation, a procedure that reopens the vaginal introitus [22]. Deinfibulation is necessary for vaginal births and also can reduce sexual pain, alleviate urinary problems, and allow for easier passage of menstrual blood [23]. Without deinfibulation, first attempts at vaginal intercourse can result in extreme pain [24]. For example, participants in one qualitative study [24], reported their husbands had to push through the small vaginal opening, causing pain at each attempt. Some experienced bleeding and required medical intervention.

Deinfibulation is considered a safe and effective procedure [25]. However, there is emerging evidence that early deinfibulation (i.e., before pregnancy) may result in a higher risk of tearing at the time of childbirth [26]. Additionally, there can be cultural reasons to delay deinfibulation until after marriage, such as fear that male partners may question a woman’s virginity if deinfibulated prior to marriage [27, 28]. Despite these concerns, women report positive attitudes towards deinfibulation, often recommending it to younger family members and friends as a pain-reducing intervention [27]. Some scholars have suggested that acculturation level may also impact one’s willingness to deinfibulate [11].

## Theoretical/Conceptual Framework

There are multiple decisional elements related to deinfibulation, including (1) timing, (2) involving family members and partners in the decision, (3) incorporating cultural values, (4) obtaining reliable medical information, and (5) weighing the benefits and risks of the procedure. Somali women who have experienced FCG may face multiple risk factors for decisional conflict and exclusion from SDM. However, to our knowledge, decisional conflict regarding deinfibulation has not been examined previously. The goal of the present study was to explore decisional conflict regarding deinfibulation using self-reported experiences surrounding the deinfibulation process. To achieve this goal, several aims were addressed, including to: (1) adapt a measure of decisional conflict for use with deinfibulation, (2) explore the level of decisional conflict about deinfibulation among Somali women who migrated to Minnesota, (3) examine differences in decisional conflict between participants who were deinfibulated (a) before pregnancy or childbirth; (b) during labor and delivery; or (d) never.

## Methods

### Overview

The present study is part of a larger study on factors associated with sexual pain in women who were infibulated [24]. We utilized a community-based participatory research approach (CBPR) by engaging community partners/members throughout the study [29]. An 11-member community advisory board (CAB) composed of Somali health professionals, school administrators, and community leaders met regularly to provide input and guidance. The analyses presented here focus solely on decision making around deinfibulation. The study was approved by University of Minnesota.

## Study Participants

We used medical clinic-based, community-based convenience, and snowball sampling methods to recruit participants in the [blinded for peer review] metropolitan area from September 2021 – April 2023. Within [blinded for peer review] - the site of this study - the Somali population is over 80,000 people [17]. Recruiters approached women prior to their medical appointments at two participating medical clinics and conducted outreach recruitment in Somali community spaces (e.g., community centers). Participants were encouraged to refer friends, family members, and/or neighbors. Prior to scheduling, recruiters ensured inclusion criteria were met: (1) Somali identity, (2) between ages 18–45, (3) undergone FGC, (4) able to consent, and (5) sexually active in the previous year. Almost half of the participants were recruited by referral from another participant (*n* = 141, 47.0%), followed by health clinic recruitment (*n* = 87, 29.0%), community recruitment *(n* = 47, 15.6%), and a previous qualitative study (*n* = 25, 8.4%). All data collection was conducted in-person at the participant’s preferred location; almost all (95.3%) chose to meet in a residential space. Participants were provided $75 for participation and an additional $30 for childcare, as needed. The interviewer reviewed the English or Somali written consent form with the participant and allowed time for participants to ask questions about the study prior to signing.

## Data Collection

Demographic items were collected as part of a larger questionnaire which was administered with audio computer-assisted self-interviewing (ACASI). A structured interview was conducted to gather information about FGC history, deinfibulation, and administer the decisional conflict scale. We used a validated iterative process with watercolor-based visual images to determine type of FGC [30, 31]. Face to face interviewing, rather than ACASI, was used for questions surrounding decision making for deinfibulation to allow for greater nuance.

## Measures

All items were written at a 5th or 6th grade reading level and translated into Somali by a bilingual professional translator. We used a team translation approach, such that two bilingual community researchers on our team reviewed the translations and discussed any words requiring further clarity [32]. The English and Somali versions were reviewed through pilot testing by bilingual community members on our CAB who did not participate in the translation process.

## Demographic Items

Demographic items included standard demographic variables, refugee status, and FGC history. Participants were interviewed regarding FGC type, and interviewers pointed to all labeled parts of the vulva on each watercolor and read the descriptions (clitoral hood, clitoris, inner lips, outer lips, urethra, vaginal opening, and anus). Interviewers asked participants to describe what was modified, cut, or removed. Interviewers documented participant’s words exactly in an open text box. Based on responses, interviewers entered the FGC type best matching the full description (Table 1). Women who described being infibulated were asked questions regarding deinfibulation, including if, when, and how many times their circumcisions were opened. A small number of participants (*n* = 23, 7.7%) chose not to view the FGC watercolor images.

## Decisional Conflict Scale – Low Literacy Version

The Decisional Conflict Scale (DCS) Low Literacy Version is a validated [33] 10-item, 3-point scale assessing certainty and conflicting values about medical decisions and perceived pressure from stakeholders [8]. The scale has stem questions that researchers adapt for the specific medical decision – in this case, deinfibulation. DCS questions are focused on retrospective self-reported perspective of participants surrounding their prior decision to deinfibulate. Participants who were infibulated were administered the DCS, and interviewers adapted questions to the participants’ wording as necessary. For example, if a participant was deinfibulated and described previously having to make that decision at a certain point in time, the interviewer used the participant’s words to orient her to that point in time. Per scoring instructions [7], questions 2–10 were scored as yes = 0; unsure = 2; and no = 4; scores were summed, divided by 10, and multiplied by 25. For question 1, if participant knew all options = 0; some options = 1; unsure = 2; none = 4. Options were written by the research team and included deinfibulation (a) before sex, (b) after sex before pregnancy, (c) during pregnancy, and (d) at the time of childbirth. The total DCS score reflected the overall level of decisional conflict ranging 0-100, where 100 represents the highest level of decisional conflict. Across 37 studies of various medical decisions, the average score for total decisional conflict on the low literacy DCS is 20.1 [34]. Internal consistency has been acceptable in previous studies (α = 0.72 to 0.86) and was excellent for the total score in the present study (α = 0.91). The DCS includes four subscales: Knowledge/Information (e.g., “did you know your options?” α = 0.75), Uncertainty (e.g., “were you clear about the best choice for you?” α = 0.87), Values Clarity (e.g., “were you clear about what mattered to you?” α = 0.86), and Support (e.g., “did you have enough support from medical professionals?” α = 0.93). Higher scores on these subscales corresponded to higher levels of decisional conflict.

## Analysis

Frequency and descriptive information were obtained for all demographic variables, DCS questions, and DCS scores. Differences in total DCS subscale and total scores by deinfibulation timing were examined with one-way ANOVAs and Bonferroni post-hoc comparisons. All analyses were conducted using SPSS version 29.0.1.0.

## Results

### Participants

Participants (*n* = 300) identified as Somali women. The most common FGC type was infibulation (Type 3; *n* = 198; 66%). Of those infibulated, 143 individuals (72%), were deinfibulated at least one time, most commonly during childbirth (46%). Demographic information is presented in Table 2. There was very little missing data in this sample; 3–4% of data for each subscale of the outcome variables were missing. Thus, we used listwise deletion to manage missing data.

### Decisional Conflict Scale

Table 3 displays the DCS items and response frequencies, including item frequency by deinfibulation history. Due to small cell sizes, tests of significant differences by deinfibulation history were not conducted. The majority of participants did not have a comprehensive understanding of all their deinfibulation options. Notably, while about two-thirds of participants indicated confidence in their choice, there still remained significant uncertainty even after the decision was made. Over one-third of individuals reported they did not get enough advice from their healthcare provider regarding deinfibulation; similarly, almost one-third felt pressured by HCPs about their decision (Table 3). Responses to these items indicate potential lack of accurate and complete knowledge regarding deinfibulation, and lingering uncertainty after the decision was made. Overall, participants reported greater decisional conflict across domains than has been found in similar studies (Table 4). For reference, the average subscale scores across 37 studies ranged from 22.0 to 26.1 [34]. The DCS total and subscale scores were normally distributed with skewness and kurtosis values falling within the accepted range of normality.

### Factors Associated with DCS Scores

The DCS Total Score was not associated with potential covariates, including age at immigration, years spent in the USA, refugee status, English speaking ability, education, or income. Differences in decisional conflict (DCS Total and subscale scores) by deinfibulation timing (never, before pregnancy, or during pregnancy and childbirth) were tested with one-way ANOVAs and Bonferroni post-hoc comparisons (Tables 5 and 6). The DCS total score significantly differed by deinfibulation timing (*p* = .009, η^2^=0.05, small effect). Significant differences in DCS total score were observed between those who were never deinfibulated (*M* = 43.40*)* and those deinfibulated during pregnancy and childbirth (*M* = 24.07; *p*=.011; *d* = 0.58, medium effect). Similarly the DCS Uncertainty subscale scores significantly differed by deinfibulation timing (*p*=.004; η^2^=0.06, medium effect), with those who were never deinfibulated scoring higher (*M* = 49.43) than those deinfibulated during pregnancy and childbirth (*M* = 22.78; *p*=.003; d = 0.63, medium effect). Finally, Support subscale scores significantly differed by deinfibulation timing (*p*<.001; η^2^=0.14, large effect), with those who were never deinfibulated scoring significantly higher (*M* = 58.91) than participants deinfibulated before pregnancy (*M* = 33.99; *p*=.006; *d*=0.59, medium effect) and during pregnancy or childbirth (*M* = 20.19; *p*<.001; *d* = 1.07, large effect). In each statistically significant comparison, the never deinfibulated group had the highest decisional conflict score (i.e., more conflict), and the during pregnancy/child-birth group had the lowest decisional conflict score.

## Discussion

Our findings demonstrated that Somali women in Minnesota who have experienced infibulation experienced some degree of decisional conflict regarding deinfibulation at the time they were making the decision. Further, this decisional conflict was higher than the average decisional conflict for other medical decisions across 37 studies [34]. The group that never underwent deinfibulation had the most overall decisional conflict, with significantly higher Uncertainty and Support subscores compared to those who chose to deinfibulate. Furthermore, effect sizes were medium to large for all significant findings, indicating further clinical importance. Thus, patients who were never deinfibulated were less likely to have the knowledge and support needed to confidently make a decision about deinfibulation compared to participants that underwent the procedure. This lack of knowledge and support is likely augmented by cultural and religious factors such as stigma against discontinuing FGC in some communities, religious pressures to continue the practice, and gender dynamics that may increase hesitancy in pursuing deinfibulation [35]. Yet, recent research in the Somali diaspora community suggests that Somali women may have lacked knowledge early in their lives, but after deinfibulation, they strongly supported other women being deinfibulated [27].

To our knowledge, no published studies have examined decisional conflict or shared decision making (SDM) among patients with FGC pursuing deinfibulation. However, moderate levels of decisional conflict regarding other pregnancy-related health decisions [6], including amongst pregnant patients considering general prenatal decisions [34] or further prenatal testing for fetal chromosomal abnormalities [36] have been reported in scientific literature.

### Gynecologic and Obstetric Decision Making

Applying SDM principles and decision support interventions (DESIs) have been found to reduce decisional conflict. For example, a systematic review evaluating DESIs in pregnancy-related decisions found a significant reduction of decisional conflict when decisional aid tools were used [2]. Another study found that the largest improvement in DCS after DESI implementation was in decision makers who were ill or made decisions for themselves [37]. In one study, mothers were surer about their choices for postpartum mother-infant care with an SDM approach, more confident regarding the risks and benefits of each option, and felt supported by their HCP [7]. Even when SDM strategies are implemented, individuals in vulnerable groups are less likely to report experiencing inclusive decision-making [7]. Comprehensive implementation of SDM principles in FGC patients, particularly in vulnerable populations, could improve decisional conflict in all DCS subscales.

In the Somali-American population specifically, SDM must be cognizant of cultural, familial, and social factors that contribute to the FGM/C decision making process. For example, Somali male partners have been shown to generally not have significant support for FGC, but report significant aversion to discussions of pregnancy and delivery, limiting their participation in deinfibulation decisions. Somali men also perceive HCP as being unprepared to care for women with FGC [38]. The decision to infibulate, as well as deinfibulate, is a strongly matriarchally guided practice, with mothers who believed in the practice being more likely to consent to FGC for their daughters [39]. SDM principles must be applied with these cultural factors in mind, in discussion with family members that the patient consents to participate. These cross-cultural considerations will help Somali women reach a decision regarding deinfibulation with more certainty, decreasing decisional conflict and decisional regret.

### Provider Preparedness in FGC Healthcare

A large barrier to advancement in FGC healthcare is lack of provider preparedness. If providers do not feel equipped with the necessary tools and knowledge to address FGC issues, dissemination of information and trust building with patients are compromised. In North American studies, 50% – 79% of relevant HCPs report an overall lack of knowledge and preparedness for caring for patients with FGC [40, 41]. In another study, an attitude assessment revealed lack of familiarity among HCPs regarding the cultural context of FGC and current laws surrounding the practice [42, 43].

### Clinical and Research Implications

There is a strong need for HCPs to discuss deinfibulation comprehensively in primary care. Our results indicate the need for improvement in our healthcare system’s ability to deliver accurate, understandable, and comprehensive education regarding deinfibulation options, risks, and benefits. Integrating FGC healthcare discussions earlier in the primary care relationship allows for a longitudinal discussion, with an initial introduction into deinfibulation followed by ample opportunity for patients to ask questions and learn more about the risks and benefits as their needs evolve. Integration of SDM in this process would involve the use of informational aids and tools to measure patients’ level of understanding of the risks and benefits of deinfibulation. A lack of clinical knowledge about FGC affects a provider’s capacity to offer clinical guidance on deinfibulation, and may contribute to negative attitudes and discomfort treating patients with FGC. These concerns could disrupt patient trust in the healthcare system and impact FGC care and treatment planning. The use of provider training guides, such as the one released by the WHO [44], have been found to successfully address provider gaps in knowledge [45]. Greater research into the efficacy of these interventions is needed before these practices are integrated into broader clinical practice.

This study demonstrated that the DCS scale can be utilized to investigate deinfibulation decisional conflict. Future studies on this topic should focus on analyzing the efficacy of decisional aid interventions and increased provider training on decisional conflict regarding deinfibulation.

### Strengths and Limitations

While this study was statistically powered for relevant analysis, larger sample sizes for deinfibulation history would allow for further between-group analysis. For example, few individuals in the present study were deinfibulated prior to pregnancy/labor, resulting in insufficient power for analyses with this subgroup. It is important to note that sampling for this study was done via community engagement, which could lead to sampling bias and homogeneity (e.g., education or healthcare access level). However, the use of community engagement to recruit participants and an in-person interview may contribute to an increased personal connection leading to authentic discussions by participants about sensitive subjects and increased participant candor.

## Conclusion

This study analyzed decisional conflict amongst Somali participants with FGC regarding deinfibulation medical decision making. We found that decisional conflict regarding deinfibulation was associated with a lack of comprehensive knowledge of the procedure itself and perceived lack of HCP support. Longitudinal studies analyzing the impact of interventions intended to reduce decisional conflict would provide strong insight into the most effective strategies to address this health concern. Increased provider training, implementing SDM principles within the appropriate cultural context, and the integration of DESIs could reduce knowledge gaps, increase trust in HCPs, and empower FGM/C patients to determine if deinfibulation is right for them.

## Figures and Tables

**Table 1:** Female genital cutting/mutilation types: world health organization classification

Type	Description
Type I	Partial or total removal of the clitoral glans and/or prepuce/clitoral hood.
Type II	Partial or total removal of the clitoral glans and the labia minora, with or without removal of the labia majora.
Type III	Narrowing of the vaginal opening with a covering seal, formed by repositioning the labia minora or labia majora, with or without removal of the clitoral prepuce/ clitoral hood and glans.
Type IV	All other harmful procedures to the female genitalia for non-medical purposes, such as pricking, piercing, incising, scraping, and cauterization.

** Definitions are from the WHO Classifications (WHO, 2008)

**Table 2:** Participant demographics (*n* = 300): numbers, percentages, means and standard deviations

Variable	*n*	%	M	SD
Age (*n* = 300)	300		36.59	5.43
*Education (n = 298)*				
No school/ESL Class	46	15.4		
Elementary school/Some high school	138	46.3		
High school diploma	58	35.6		
Some college/Associate’s degree	41	13.8		
Bachelor’s degree/Master’s degree	15	5.3		
*Employment status (n = 300)*				
Part-time	138	46.0		
Full-time	64	21.3		
Homemaker	65	21.7		
Unemployed/Disabled/ Other	33	11.0		
Years in the U.S. (*n* = 300)	300		14.18	6.72
Age at Immigration (*n* = 300)	300		22.41	7.04
Stayed in Refugee Camp (*n* = 298)s	157	52.7		
Language spoken: Somali (*n* = 299)	257	86.0		
Language spoken: English (*n* = 299)	197	65.9		
*Household Income (n = 283)*				
Less than $15,000	61	21.6		
$15,000 - $30,000	123	43.5		
$30,000 - $45,000	61	21.6		
$45,000 - $60,000	25	8.8		
Greater than $60,000	13	4.6		
*FGC Type (n = 300)*				
Type 1	80	26.7		
Type 2	19	6.3		
Type 3	198	66.0		
Type 4	2	0.7		
Type 1 or 2	1	0.3		
*Deinfibulation (n = 189)*				
Never	47	23.7		
Before pregnancy	51	25.7		
During childbirth/pregnancy	91	46.0		

**Table 3:** Decisional conflict scale survey questions results

Deinfibulation History								
Question	Never		Before Pregnancy/Childbirth		During Pregnancy/Childbirth		Total	
	*n*	%	*n*	%	*n*	%	*n*	%
*Informed Subscore* 1. Did you know about your options for opening your circumcision? * (*n* = 183) All options	14	33.2	9	17.6	19	21.1	42	23.0
Some options	17	40.5	30	58.8	66	73.3	113	61.7
Unsure**	11	26.2	12	5.6	5	5.6	28	15.3
2. Do you know the benefits of opening your circumcision? (*n* = 186) Yes	28	60.1	33	64.7	62	69.7	123	66.1
No	18	39.9	17	33.3	25	28.1	60	32.3
Unsure	0	0	1	2.0	2	2.2	3	1.6
3. Do you know the risks and side effects of opening your circumcision? (*n* = 186) Yes	25	55.6	21	41.2	58	64.4	104	56.0
No	20	44.4	29	56.9	29	32.3	78	42.0
Unsure	0	0	1	1.9	3	3.3	4	2.0
*Values Clarity Subscore* 4. Do you know which benefits of deinfibulation matter most to you? (*n* = 185) Yes	27	60.0	33	66.0	71	78.9	131	70.9
No	17	37.8	17	34.0	17	18.9	51	27.6
Unsure	1	2.2	0	0.0	2	2.2	3	1.6
5. Do you know which risks and side effects of deinfibulation matter most to you? (*n* = 186) Yes	30	66.7	26	51.0	63	70.0	119	64.0
No	15	33.3	24	47.0	24	26.7	63	33.9
Unsure	0	0	1	2.0	3	3.3	4	2.1
*Support Subscore* 6. Do you feel you received enough support from your HCP*** to make a choice? (*n* = 185) Yes	16	36.4	35	68.6	76	84.4	127	68.6
No	26	59.1	15	29.4	13	14.4	4	21.6
Unsure	2	4.5	1	2.0	1	1.1	54	29.2
7. Do you feel that your HCP did not pressure you regarding your decision to make a choice? (*n* = 184) Yes	20	46.5	30	58.8	67	74.4	117	63.6
No	20	46.5	19	37.2	20	22.2	59	32.1
Unsure	3	7.0	2	4.0	3	3.3	8	4.3
8. Do you feel you got enough advice from your HCP regarding your decision to deinfibulate? (*n* = 184) Yes	14	32.6	34	66.7	70	77.8	118	63.8
No	28	65.1	16	31.3	19	21.1	64	34.6
Unsure	1	2.3	1	2.0	1	1.1	3	1.6
*Uncertainty Subscore* 9. Do you feel clear about the best choice for you? (*n* = 185) Yes	21	47.7	36	70.6	70	77.8	127	68.6
No	22	50.0	13	25.5	18	20.0	53	28.6
Unsure	1	2.3	2	3.9	2	2.2	5	2.7
10. Are you sure about your decision? (*n* = 185) Yes	22	50.0	33	64.7	67	74.4	122	65.9
No	20	45.5	16	31.3	21	23.3	57	30.8
Unsure	2	4.5	2	3.9	2	2.2	6	3.2

** Unsure was an option for decision making conflict questions; If they chose none of the options, they are missing data. Anyone eligible for deinfibulation was coded as yes, no, or unknown.

*** HCP = Healthcare Provider.

**Table 4:** Decisional conflict scale results for participants undergoing deinfibulation

Variable	M	SD
Total Score (*n* = 180)	32.35	35.49
Uncertainty Subscore (*n =* 190)	32.24	44.43
Knowledge/Information Subscore (*n =* 183)	33.06	32.02
Values Clarity Subscore (*n =* 190)	32.63	43.65
Support Subscore (*n =* 189)	33.86	41.15
Range = 1–100		

**Table 5:** DCS Subscores and Deinfibulation Timing: Comparison with ANOVA

			Deinfibulation Timing					
			Before pregnancy		During pregnancy/childbirth		Never	

	*F*	*^p^*	*M*	*SE*	*M*	*SE*	*M*	*SE*
Uncertainty	5.69	≤ 0.01	30.39	6.16	22.78	4.21	49.43	7.20
Knowledge/Information	2.12	0.12	39.54	4.41	28.19	3.06	32.93	5.70
Values Clarity	2.80	0.06	41.00	6.34	24.17	4.17	36.11	7.00
Support	15.03	≤ 0.001	33.99	5.96	20.19	3.49	58.91	6.45
DCS Total Score	4.89	0.01	36.10	5.16	24.07	3.31	43.40	6.06

F is reported as * ≤0.05; ** ≤ 0.01; *** ≤ 0.001. Statistical significance for post-hoc analyses are reported in-text.

**Table 6:** Post-Hoc comparison with Bonferroni

	*Deinfibulation* *Timing (I)*	*Deinfibulation Timing (J)*	*Mean* *Difference*	*SE*	*P*
DCS Total Score	*Before Pregnancy*	*During Pregnancy*	5.58	7.15	1.00
		*Never*	16.90	6.43	0.03
	*During Pregnancy*	*Never*	11.32	5.91	0.17
			
Uncertainty	*Before Pregnancy*	*During Pregnancy*	18.08	9.18	0.15
		*Never*	25.04	8.25	0.01
	*During Pregnancy*	*Never*	6.97	7.60	1.00
			
Knowledge/Information	*Before Pregnancy*	*During Pregnancy*	−12.36	6.83	0.22
		*Never*	−3.57	6.14	1.00
	*During Pregnancy*	*Never*	8.79	5.65	0.37
			
Values Clarity	*Before Pregnancy*	*During Pregnancy*	−7.03	9.10	1.00
		*Never*	9.54	8.18	0.74
	*During Pregnancy*	*Never*	16.56	7.53	0.01
			
Support	*Before Pregnancy*	*During Pregnancy*	23.60	8.14	0.01
		*Never*	36.85	7.31	0.00
	*During Pregnancy*	*Never*	13.25	6.73	0.15
			

## Data Availability

No datasets were generated or analysed during the current study.
